# The Amyloid Precursor Protein—A Novel Player within the Molecular Array of Presynaptic Nanomachines

**DOI:** 10.3389/fnsyn.2015.00021

**Published:** 2016-01-20

**Authors:** Melanie Laßek, Jens Weingarten, Martin Wegner, Walter Volknandt

**Affiliations:** ^1^Department of Molecular and Cellular Neurobiology, Goethe University FrankfurtFrankfurt am Main, Germany; ^2^Department of Molecular Bioinformatics, Goethe University FrankfurtFrankfurt am Main, Germany

**Keywords:** Alzheimer’s disease, amyloid precursor protein, calcium homeostasis, mitochondria, presynaptic active zone

## Abstract

More than 20 years ago the amyloid precursor protein (APP) was identified as the precursor protein of the Aβ peptide, the main component of senile plaques in brains affected by Alzheimer’s disease (AD). The pathophysiology of AD, characterized by a massive loss of synapses, cognitive decline, and behavioral changes was in principle attributed to the accumulation of Aβ. Within the last decades, much effort has gone into understanding the molecular basis of the progression of AD. However, little is known about the actual physiological function of APPs. Allocating APP to the proteome of the structurally and functionally dynamic presynaptic active zone (PAZ) highlights APP as a hitherto unknown player within the setting of the presynapse. The molecular array of presynaptic nanomachines comprising the life cycle of synaptic vesicles, exo- and endocytosis, cytoskeletal rearrangements, and mitochondrial activity provides a balance between structural and functional maintenance and diversity. The generation of genetically designed mouse models further deciphered APP as an essential player in synapse formation and plasticity. Deletion of APP causes an age-dependent phenotype: while younger mice revealed almost no physiological impairments, this condition was changed in the elderly mice. Interestingly, the proteomic composition of neurotransmitter release sites already revealed substantial changes at young age. These changes point to a network that incorporates APP into a cluster of nanomachines. Currently, the underlying mechanism of how APP acts within these machines is still elusive. Within the scope of this review, we shall construct a network of APP interaction partners within the PAZ. Furthermore, we intend to outline how deletion of APP affects this network during space and time leading to impairments in learning and memory. These alterations may provide a molecular link to the pathogenesis of AD and the physiological function of APP in the central nervous system.

## APP—Networking within Presynaptic Nanomachines (Introduction)

The idea of nanotechnology helping to redesign everything at the atomic level goes back to the American engineer Eric Drexler (Drexler, 1987). More than 20 years ago, he published his still controversially discussed book “Engines of Creation” almost at the same time as scientists discovered a protein that fits perfectly in the conceptual idea of nanotechnology. The protein, named amyloid precursor protein (APP), was originally discovered as precursor of amyloid beta (Aβ), the main component of senile plaques and hallmark of Alzheimer’s disease (AD; Glenner and Wong, 1984; Kang et al., 1987). Nanomachines are autonomic miniature machines that can work on their own. However, under distinct circumstances they demand on helping hands called assemblers. The vision of autonomic assemblers performing every physical and chemical application, that can have a lasting positive but also negative effect on people’s life, would change the way of scientific thinking in a sustainable manner (Drexler, 1987). If we transpose this image to APP, our protein of interest, we can observe similar properties in regulating synaptic development and degeneration. Enzymatic processing, ligand-binding, and dimerization of APP can induce the development and maintenance of neuronal circuits but also their degeneration. Aβ, mainly associated with the pathogenesis of AD can also protect neurons from neurotoxicity. Furthermore, elimination of synapses initiated by Aβ already occurs during the development of neuronal circuits (Kamenetz et al., 2003; Hsieh et al., 2006; Abrahamsson et al., 2007; Wasling et al., 2009). This sensitive balance between physiological benefit and pathophysiological hallmarks is reflected by many processes taking place within a synapse.

Regarding the synapse at the macroscopic level, there are two highly complex nanomachines named pre- and postsynaptic terminal. However, on a microscopic level, both termini can be further subdivided into more restricted nanomachines with specific tasks and assemblers that regulate and control their functions (e. g., neurotransmitter release, signal transduction and reorganization). Within this review, we will focus on the presynaptic terminal with special emphasis on the neurotransmitter release site and its constituent the APP.

Allocating APP to the proteome of the structurally and functionally dynamic presynaptic active zone (PAZ) identified APP as a hitherto unknown player within the setting of the presynaptic nanomachines (Laßek et al., 2013). The molecular array of presynaptic nanomachines comprising the life cycle of synaptic vesicles, exo- and endocytosis, cytoskeletal rearrangements, and mitochondrial activity provides a balance between structural and functional maintenance and diversity (Südhof, 2012; Laßek et al. 2014b, 2015; Weingarten et al., 2014, 2015). The generation of genetically designed mouse models further deciphered APP as an essential player in synapse formation and plasticity (Heber et al., 2000; Wang et al., 2005, 2009b; Ring et al., 2007; Weyer et al., 2011; Hick et al., 2015). Deletion of APP causes an age-dependent phenotype: while younger mice revealed almost no physiological impairments, this condition was changed in the elderly mice (Phinney et al., 1999; Priller et al., 2006; Ring et al., 2007). Interestingly, substantial changes of the proteomic composition of neurotransmitter release sites are already detectable in younger mice (Laßek et al., 2014a). Since APP plays an essential role during the development of neuronal circuits, it was suggested that the amyloid precursor like protein APLP2 compensates for the loss of APP (Weyer et al., 2011; Hick et al., 2015). Therefore, it is tempting to speculate that APP itself can act as a nanomachine with its assembler APLP2. The deletion of the assembler does not account for a severe phenotype. However, at the protein level the abundance of the nanomachine APP becomes increased (Laßek et al., 2014a). Reversely, deletion of the APP can only be compensated by the assembler up to a certain time. Like an electronic device that still works after removing from its charger until the battery has discharged.

**Figure 1:**
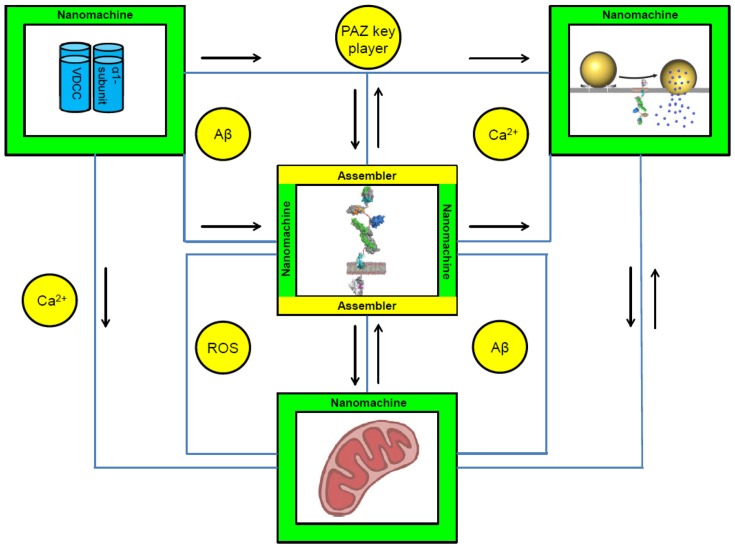
**Conceptual circuit integrating nanomachines and assemblers within the presynaptic active zone (PAZ)**. APP is embedded into the circuit of nanomachines within the PAZ. It can act within different physiological processes at the presynaptic terminal including synaptic vesicle exocytosis, Ca^2+^-homeostasis and mitochondrial function. A well balanced proportion between the machines and their assemblers is the prerequisite for the development and maintenance of the neuronal circuits. Nanomachines are visualized as green squares; assemblers as yellow circles. APP can act as a nanomachine and as an assembler—the square is therefore colored in green and yellow. Black arrows represent the interactions between individual nanomachines. Connecting lines are highlighted in blue. Depicted are: α-subunit of voltage dependent Ca^2+^-channels (VDCC); exocytosis of SV, with APP embedded into the presynaptic plasma membrane; illustration of APP; mitochondria. Abbreviations: Aβ, amyloid-beta; Ca^2+^, calcium ion; PAZ, presynaptic active zone; ROS, reactive oxygen species.

Within the presynapse many clusters of nanomachines could be identified. These machines are involved in numerous physiological processes that have to be regulated, coordinated and modified. In the following sections APP will be embedded into selected presynaptic clusters of nanomachines (Figure 1).

## APP and Synaptic Vesicle Exocytosis—Assemblers at Work

Synaptic transmission requires a coordinated network decoding an action potential into a chemical signal at the PAZ. Therefore, consecutive steps including the recruitment of Ca^2+^-channels, exocytosis and endocytosis are processed by nanomachines and its assemblers. The recruitment of Ca^2+^-channels as nanomachines is performed by a protein complex comprising the assemblers RIM, Munc13, RIM-BP, α-liprin and CAST-proteins. These assemblers belong to the so called “active zone key proteins”, also including bassoon, ELKS and CASK proteins. APP as an assembler is functionally integrated within this conserved network of active zone key players (Figure 1). The interaction with bassoon regulates the recruitment of ERC protein 2 (ELKS), RIM, and Munc13 that are essential for docking and priming of synaptic vesicles (Südhof and Rizo, 2011; Südhof, 2012, 2013b).

Exocytosis of synaptic vesicles is mediated by SNARE-complex formation whereby the integral synaptic vesicle protein VAMP2 forms a complex with the presynaptic plasma membrane proteins syntaxin-1 and SNAP25. The SNARE complex is a nanomachine that is characterized by a quadruple α-helix bundle with VAMP2 and syntaxin-1 contributing one α-helix each and SNAP25 contributing two α-helixes. Before the SNARE complex can be formed, syntaxin-1 has to change its conformation from closed (due to binding to the assembler Munc-18); to open (supported by the assembler Munc13). In the open conformation, Munc-18 is still associated with syntaxin-1 supporting the interaction between SNARE motifs derived from syntaxin-1 and SNAP25 (Südhof, 2013b). The resulting donor-complex further binds to VAMP2 assembling to a Munc-18-SNARE complex, with Munc-18 being supposed to mediate interactions between the four-helix bundle and the plasma membrane necessary for membrane fusion (Deák et al., 2009). Furthermore, Munc-18 does not detach during the SNARE complex assembly/disassembly cycle, emphasizing its essential role in exocytosis (Südhof, 2013a). APP plays a crucial role in synaptic vesicle exocytosis and further emphasizes the compensatory role of APLP2 in APP-KO mice. Moreover, the interaction of APP with presynaptic proteins involved in the regulation of exocytosis and the putative function in tuning this process is important to unravel the physiology of APP within the CNS (Fanutza et al., 2015).

Within the molecular nanomachinery of SNARE-complex formation, α-synuclein is an indispensable assembler. The role of α-synuclein during SNARE complex formation is the implementation of VAMP2 into the SNARE donor complex (Burré et al., 2010). Therefore, the interaction between synaptophysin and VAMP2 has to be repealed before complete vesicle fusion can take place (Valtorta et al., 2004) putatively mediated by α-synuclein. At protein level, deletion of α-synuclein does not alter the abundance of SNARE proteins, but diminishes the ability of SNARE complex formation. In terms of nanotechnology, loss of the assembler accounts for severe impairments of the nanomachine. Interestingly, α-synuclein and APP are both assemblers within this highly complex SNARE-machinery, but they act differently. While α-synuclein mediates SNARE-complex formation, APP might execute a regulatory function during docking and priming of synaptic vesicles. After neurotransmitter release, the assembler NSF mediates the hydrolysis of ATP and triggers the disassembling of the SNARE nanomachine (Littleton et al., 2001).

## APP and Calcium—Partners in Crime

During the mid-1980’s when APP was discovered as precursor protein of Aβ, another crucial player within the pathogenesis of AD was identified—calcium (Khachaturian, 1987; Landfield, 1987; Landfield et al., 1989; Small et al., 2009). APP and calcium share some interesting features that interconnect them as partners in crime. Moreover, they fit perfectly into the conceptual idea of nanomachines and assemblers. They can operate as neurotrophic assemblers supporting proper physiological function of the presynaptic terminal, but they also trigger neurodegeneration.

Ca^2+^-influx through voltage-dependent channels within the presynaptic terminal is essential for neurotransmitter release (Mattson, 2007). The complex protein machinery (Südhof, 2012) that ensures the presence of voltage dependent calcium channels (VDCC) at active zones after the arrival of action potentials comprises a varying set of assemblers (Figure 1). These assemblers have to act in a consecutive manner to provide a rapid increase in intracellular Ca^2+^. However, the elevation of intracellular Ca^2+^-levels is only transiently and residual amounts of Ca^2+^ have to be removed quickly to avoid adverse effects on neurons (Mattson, 2007; Bezprozvanny and Mattson, 2008; Small et al., 2009).

Disruption of Ca^2+^-homeostasis can account for neuronal dysfunction and neurodegeneration. In this context, Aβ oligomers have been discussed to induce increase intracellular Ca^2+^-levels, thereby altering synaptic signaling and changing the activity of neighboring neurons. Since neuronal activity is under the control of a neuronal network, systematic failure within this network account for alterations within the network circuit (Small, 2008; Small et al., 2009). The vicious cycle describing the maintenance of the neuronal signaling within the network is based on synaptic compensation (scaling). Aβ oligomers induces elevated Ca^2+^-influx, that accounts for synaptic dysfunction, followed by synaptic scaling and consequently increased excitability with in turn leads to elevated intracellular Ca^2+^. At the end, healthy neurons degenerate due to Ca^2+^-dysregulation and synaptic dysfunction (Small, 2008). Interestingly, the abundance of VDCC, in particular L-type calcium channels (LTCC), is also increased during aging and in AD (Thibault and Landfield, 1996; Thibault et al., 2001).

APP is involved in the recruitment of VDCC (Figure 1) and the regulation of their abundance at hippocampal neurotransmitter release sites. The hippocampus, a brain region of interest regarding learning and memory consolidation, is highly susceptible for excitotoxicity and neurodegeneration. Both can be induced and triggered by long-lasting elevated intracellular Ca^2+^-levels. Moreover, diminished endocytosis of VDCC in the absence of APP accounts for a dysregulation of the balance between inhibitory and excitatory neurons. GABAergic hippocampal neurons revealed increased activity due to an increased abundance of VDCC at presynaptic terminals (Yang et al., 2009). Moreover, LTP deficits in APP double and single mutant mice were rescued by application of GABA_A_ receptor inhibitor picrotoxin (Fitzjohn et al., 2000; Weyer et al., 2011). This molecular interpretation of changes in calcium homeostasis and subsequent alterations in synaptic plasticity was manifested by electrophysiological network analysis. In this context, it was proposed that APP deletion induces an altered neuronal excitation-inhibition ratio (Korte et al., 2012). Within the hippocampus memory formation is based on a variety of synchronized network oscillations that are regularly synchronized between the CA1 and CA3 region (Korte et al., 2012). As inhibitory interneurons play an essential role in synchronizing these oscillations they can affect a large population of pyramidal neurons, inhibit specific input pathways and guarantee for a high background-to-noise ratio (Mann and Paulsen, 2007). This might explain the reported alteration in the excitation-inhibition ratio (Korte et al., 2012). Similarly, alterations during the pathogenesis of AD such as changes in personality, sleep disturbance and changes in awareness could be traced back to a dysregulation of the glutamate-GABA metabolism (Robinson, 2000; Doert et al., 2015). GABA can be produced by the so called GABA shunt, a bypass mechanism that skips the intra-mitochondrial α-ketoglutarate-dehydrogenase (Mamelak, 2012). This mechanism becomes operative when the metabolism of glutamate is enhanced probably due to reduced glutamine synthase activity. Elevated GABA levels in transgenic APP mutant mice, carrying the Swedish and London mutation for APP, (Doert et al., 2015) reflect a situation for the glutamate-GABA ratio similar to that observed for APP knockout mice (Weyer et al., 2011; Korte et al., 2012). Both phenotypes indicate a critical role of APP in memory formation and consolidation as well as behavioral aspects that are severely affected in in the respective mutants and in AD patients (Ring et al., 2007; Doert et al., 2015).

## APP and Mitochondria—Nanomachines and Assemblers Two-In-One

Within the presynaptic terminal, mitochondria are essential nanomachines, providing energy supply and calcium buffering for a large variety of physiological functions. However, mitochondrial dysfunctions are in focus of playing an important role in the pathology of AD.

Considering the pros and cons of nanomachines at work as described above for APP and Aβ, we can outline a similar feature for mitochondria that can be physiological but also pathophysiological. Both subjects can function as nanomachine and assembler depending on the perspective. Mitochondria produce reactive oxygen species (ROS) that are assemblers (Figure 1). The amount of ROS is in general regulated by oxygen donor concentration and enzymes of the electron transport chain. Oxygen level and donor concentration trigger the amount of ROS in a linear manner. ROS, produced as superoxide anion (O_2_^•−^), hydrogen peroxide (H_2_O_2_) and hydroxyl radical (^•^OH) at complex I and complex III of the electron transport chain need to be critically regulated (Leuner et al., 2012a). Within the mitochondrial matrix ROS production depends critically on the proton gradient (Δp), the NADH/NAD^+^ and CoQH_2_/CoQ ratios and the local O_2_ concentration. Superoxide anions are mainly produced at complex I. Under physiological conditions, the production of O_2_^•−^ at complex III is insignificant as compared to the production rates by complex I (Murphy, 2009). Therefore, the outcome “oxidative stress” is on the one hand initiated by the mitochondria themselves, but at the same time affects its producer as first objective (Harper et al., 2004; Korge et al., 2008; Poyton et al., 2009; Müller et al., 2010). Besides ROS, dysfunction of the entire respiratory system, associated by a decrease in mitochondrial membrane potential and reduced levels of ATP, account for impairments in mitochondrial function and trigger the early onset of neurodegeneration (Rhein et al., 2009; Wang et al., 2009a; Leuner et al., 2012a). APP and Aβ have been directly associated with mitochondrial function (Leuner et al., 2010, 2012a; Eckert et al., 2012) and in this context, it would be of great interest to study the effect of APP deletion on mitochondrial function at neurotransmitter release sites.

Neuronal communication is based on reliable functioning of synaptic vesicle exo- and endocytosis. Already slight alterations can disrupt this procedure. In terms of our topic nanomachines and its assemblers, APP and mitochondria both provide a clear picture of a classical nanomachine and their proteolytic and/or metabolic products are assemblers (Figure 1). The balance between physiological benefit and pathophysiological effect is fragile. Similar to the sensitive Ca^2+^-homeostasis, imbalance between ROS and/or Aβ causes mitochondrial dysfunction, membrane protein and membrane lipid modifications (Eckert et al., 2003). Generation of ROS occurs along the electron transport chain at complex I and complex III. Recently, Leuner et al. (2012b) identified complex I in conjunction with elevated ROS levels as starting point for mitochondrial dysfunctions and onset of the amyloidogenic cascade in AD. ROS can trigger the production of Aβ via enhancing the enzymatic activity of BACE1 and γ-secretase. Interestingly, Aβ oligomers, as well as Aβ fibrils can account for a decrease in mitochondrial membrane potential and ATP levels. This phenotype has already been detected in young transgenic mice prior to Aβ plaque formation (Leuner et al., 2012a, b). Conversely, patients with mitochondrial disorders like mitochondrial encephalopathy, lactic acidosis, stroke-like episodes (MELAS) show cognitive impairments, behavioral decline and AD-like plaque formation in the absence of familiar AD evidence (Kaido et al., 1996).

It is widely accepted that changes in the interplay between APP, Aβ and mitochondrial function are likely to correlate with the onset of neurodegenerative diseases (Eckert et al., 2012; Leuner et al., 2012a). Therefore, novel therapeutic strategies that account for mitochondrial protection will be a promising approach for AD treatment or prevention.

## Conclusion and Outlook

Assemblers and nanomachines execute different but synergistic functions within the PAZ. By communicating with individual nanomachines, assemblers can control, navigate and trigger physiological functions at the presynaptic terminal. Their operations are based on an appropriate balance accompanied by a variety of interaction- and combination possibilities (e. g., SNARE complex formation and Ca^2+^-signaling). If this balance is dysregulated (e. g., increase in Aβ, ROS or calcium) and extend physiological compensation mechanisms, pathophysiological hallmarks (senile plaque formation, mitochondrial dysfunction and neurodegeneration) will increase alarmingly.

One important player within this conceptual idea of nanomachines is APP. Within the molecular array of presynaptic nanomachines APP can equally act as an individual nanomachine and assembler at once. It participates in crucial steps like synaptic vesicle exocytosis and Ca^2+^-homeostasis and has a major impact on proper mitochondrial function. All these interplays depend on a highly coordinated proteinaceous network. Unravelling how APP is embedded into individual networks within neurotransmitter release sites and how disturbances of the network may account for neurodegenerative diseases needs to be addressed in future studies. Until now, data derived from APP mutant mice (transgenic Alzheimer mouse models or knockout mouse models) clearly point to an essential role of APP in synaptic development, function and plasticity. Changes at the behavioral level (e. g., cognitive decline, impairments in LTP and memory) are characteristic for all APP mutants and reveal a time depended development.

One possibility to address the question how APP is embedded into the presynaptic nanomaschine is provided by bioinformatic tools. The mathematical field of graph theory allows the integration of all possible physical and functional relations between proteins into a single model. This model is called a protein-protein interaction network (PPI). By analyzing the topological properties of such a PPI network the key molecular players are identified and classified. These properties are used to assign a topological role to a protein of interest. Whether a protein is a highly connected hub or a less connected linker between functional modules is indicated by its topological properties in the PPI network and adds another layer of information.

The pure topological model can be augmented with additional information using, e.g., gene ontology terms and protein localizations. The understanding of the functional composition of the PAZ is facilitated by applying community detection methods. These methods detect highly connected clusters of proteins. The highly connected clusters of proteins are assumed to act within the same biological processes. By analyzing the biological functions represented by the individual communities a global picture of interconnected functions and pathways arises.

If one takes all these individual approaches together, one can design a molecular network of nanomachines and their assemblers to gain further insights into the complex physiology of presynaptic functions. Since APP affects this network during space and time, leading to the development and the loss of synapses, it is essential to understand how APP acts within these individual networks. Currently, it is not known which physiological function APP is executing but it is obvious that APP plays an indispensable role in proper synaptic function—including development and degeneration.

## Author Contributions

ML, JW, MW and WV wrote this review article.

## Conflict of Interest Statement

The authors declare that the research was conducted in the absence of any commercial or financial relationships that could be construed as a potential conflict of interest.

## Abbreviations

Aβ: amyloid beta
AD: Alzheimer’s disease
APLP2: amyloid precursor like protein 2
APP: amyloid precursor protein
CAST: CAZ-associated structural protein
CAZ: cytomatrix of the active zone
CoQ: coenzyme Q
CoQH2: reduced CoQ
LTCC: L-type calcium channels
Munc: mammalian uncoordinated
NSF: N-ethylmaleimide sensitive fusion protein
PPI: protein-protein interaction network
PAZ: presynaptic active zone
RIM: Rab-interacting molecule
RIM-BP: rab-interacting binding protein
ROS: reactive oxygen species
SNARE: soluble NSF-attachment receptor proteins
SNAP25: synaptosomal associated protein 25
VAMP2: vesicle associated membrane protein2/synaptobrevin2
VDCC: voltage dependent calcium channels.
